# Gender Bias in Leadership Roles in General Surgery: A South Asian Perspective

**DOI:** 10.7759/cureus.55900

**Published:** 2024-03-10

**Authors:** Harendra Kumar, Arkadeep Dhali, Jyotirmoy Biswas, Gopal Krishna Dhali

**Affiliations:** 1 General Surgery, Dow University of Health Sciences, Karachi, PAK; 2 Gastroenterology, University of Sheffield, Sheffield, GBR; 3 Internal Medicine, Sheffield Teaching Hospitals NHS Foundation Trust, Sheffield, GBR; 4 General Medicine, College of Medicine & Sagore Dutta Hospital, Kolkata, IND; 5 Gastroenterology, School of Digestive and Liver Diseases, Institute of Postgraduate Medical Education and Research, Kolkata, IND

**Keywords:** gender gap, south asia, general surgery, leadership, diversity

## Abstract

This article addresses the significant issue of gender bias in leadership roles within the realm of general surgery, with a particular focus on the South Asian context. The persistence of cultural norms, entrenched gender stereotypes, and discriminatory practices in this region significantly limits the opportunities available to female surgeons. It calls on all stakeholders, including medical institutions, governing bodies, and surgeons, to take an active role in eliminating gender bias and fervently supporting diversity and inclusivity in leadership positions. By doing so, it argues, we can create a more equitable and promising future for the field of general surgery in South Asia.

## Editorial

Gender bias persists as an enduring and pressing concern within the realm of leadership roles in general surgery. The upward trajectory of female representation in the surgical domain, while notable, remains plagued by persisting disparities and formidable challenges in ascending to leadership positions. Within the South Asian context, these concerns are exacerbated by the weight of cultural norms, deeply entrenched gender stereotypes, and discriminatory practices that significantly restrict the opportunities accessible to female surgeons. A survey among the Indian urology workforce highlighted discrimination in training and work, lack of mentorship, pregnancy-related compilations, and compromised careers due to family responsibilities as a few challenges faced [1].

One of the most conspicuous manifestations of gender bias emerges in the form of a stark underrepresentation of women in leadership positions. A cursory perusal of leadership boards across surgical societies, academic institutions, and healthcare facilities within South Asia lays bare an unsettling gender imbalance [2,3].

Numerous factors conjoin to fortify this gender bias, amplifying the tribulations experienced by women in surgical leadership roles. Deep-seated societal stereotypes persist, perpetuating the fallacy that surgery is an inherently masculine pursuit. These stereotypes find further reinforcement in the subtle biases pervasive in training and authorship opportunities, promotional mechanisms, and academic positions ultimately precipitating diminished confidence and mentorship for female surgeons striving to ascend the professional hierarchy. Rathna et al. discuss the authorship positions to evaluate gender disparities in the senior author positions and found that around a quarter of the articles had no female authors (23.2%) as compared to the 3.2% of the articles where there were no male authors. This can partly be explained by discrimination through something called “disparate impact”. This practice, though it can seem very fair from the outside, leads to inequality [4]. This applies to the academic as well as clinical aspects of medicine.

Another pivotal determinant amplifying gender bias within the surgical milieu is the absence of a supportive professional ecosystem. The absence of support mechanisms, encompassing adequate parental leave, accessible childcare facilities, and flexible work arrangements, serves as a discouraging counter-incentive for women to pursue leadership roles (Table 1) [5].

**Table 1:** Gender Disparities, Women Surgeons, and Leadership Positions in South Asian Countries: Challenges and Barriers

Country	Percentage of women surgeons	Percentage of women in leadership positions	Main barriers to advancement
India	7%	3%	Lack of support, harassment, unequal opportunities, sex-blindness, work-life balance, criticism [2]
Pakistan	9%	4%	Male-dominated culture, discrimination, harassment, lack of mentorship, work-life balance, family responsibilities [3]
Sri Lanka	12%	6%	Male-dominated culture, discrimination, harassment, lack of mentorship, work-life balance, family responsibilities [4]
Bangladesh	5%	2%	Male-dominated culture, discrimination, harassment, lack of mentorship, work-life balance, family responsibilities [5]

A World Health Organization report reveals a global gender disparity endemic in the health and care workforce. Although women constitute a formidable 70% of this sector, the disconcerting reality remains that they occupy merely 25% of senior positions [5]. Female surgeons in this region grapple with an intricate web of impediments on their trajectory to leadership, encompassing the absence of mentorship, the persistence of deep-seated gender stereotypes, the underpinning of cultural norms that perpetuate antiquated gender roles, and stark manifestations of discrimination.

A study conducted by the Society of American Gastrointestinal and Endoscopic Surgeons (SAGES) augments our comprehension of gender representation within the sphere of surgical leadership [2,3,6]. The study reveals that the percentage of women in leadership roles within SAGES, in comparison to the overall membership, manifests a somewhat more encouraging quotient, with no marked gender differentiation in the progression of committee members to leadership positions [2,3].

In summation, the time has arrived for the confrontation of the gender bias that has regrettably marred the trajectory of female surgeons within South Asia. Accordingly, we earnestly implore all stakeholders, encompassing medical institutions, governing bodies, and the pantheon of fellow surgeons, to assume a proactive stance in the annulment of gender bias and the zealous promotion of diversity and inclusivity in leadership positions. Together, we shall forge a path to a more equitable and promising future for general surgery within the South Asian context.
